# The epidemiology of syphilis in Ethiopia: a protocol for systematic review and meta-analysis covering the last three decades

**DOI:** 10.1186/s13643-019-1136-z

**Published:** 2019-08-22

**Authors:** Kindie Mitiku Kebede, Dejene Derseh Abateneh, Alemayehu Sayih Belay, Gizachew Ayele Manaye

**Affiliations:** 1 Department of Public Health, College of Health Sciences, Mizan-Tepi University, Tepi, Ethiopia; 2 Department of Medical Laboratory Sciences, College of Health Sciences, Mizan-Tepi University, Tepi, Ethiopia; 3 Department of Nursing, College of Health Sciences, Mizan-Tepi University, Tepi, Ethiopia; 4 Department of Medical Laboratory Sciences, College of Health Sciences, Mizan-Tepi University, Tepi, Ethiopia

**Keywords:** Syphilis, Serosyphilis, Prevalence, Seroprevalence, Magnitude, Epidemiology, Systematic review, Meta-analysis, Protocol, Ethiopia

## Abstract

**Background:**

Several individual epidemiological studies in Ethiopia suggest that syphilis is a public health problem. However, to the best of our knowledge, there is no synthesized and meta-analysis data on the epidemiology of syphilis in Ethiopia. This systematic review and meta-analysis aimed to summarize and synthesize existing data on the prevalence of syphilis in Ethiopia.

**Method:**

Studies reporting the prevalence of syphilis will be identified from major databases and gray literature. The major databases (MEDLINE/PubMed, EMBASE, Web of Science, CINAHL, The Cochrane Library, Lilacs, and African journal online) and gray literature (Google Scholar search engine, official WHO and CDC websites, the online library of academic and governmental institutions in Ethiopia) will be searched. Studies published/reported from 1 January 1990 to 1 January 2019 will be included to have a contemporary estimation. A random-effect meta-analysis of prevalence will be used after stabilizing the variance of included studies using a single arc transformation. The quality of the included studies will be assessed using the Joanna Briggs Institute Meta-Analysis of Statistics Assessment and Review Instruments. Heterogeneity and publication bias will be assessed. If significant heterogeneity is detected, subgroup analysis will be done using study region, study population, diagnostic assay/syphilis screening tool, median sample size, year of data collection, study sites, sampling method, and methodological quality as grouping variables.

**Discussion:**

This systematic review and meta-analysis intend to contribute an improved knowledge on the epidemiology of syphilis in Ethiopia. Knowledge about the epidemiology of syphilis may help policymakers and other stakeholders to allocate resources and target interventions for the prevention and elimination of syphilis.

**Systematic review registration:**

PROSPERO CRD42018116231

**Electronic supplementary material:**

The online version of this article (10.1186/s13643-019-1136-z) contains supplementary material, which is available to authorized users.

## Background

Syphilis is an infectious disease caused by the spirochaete, *Treponema pallidum*. It is one of the major public health problems causing genital laceration that may enhance the acquisition and transmission of human immunodeficiency virus (HIV) infection [1]. Vertical transmission of syphilis may cause miscarriage, stillbirth, prematurity, low birth weight, and death of the baby shortly after birth [2].

The prevalence of syphilis had declined globally over the last three decades and recent global pooled data showed that the prevalence of syphilis is 1.1%. However, the African region is consistently the most affected region having a pooled prevalence of 3.24 [3].

The decline globally may not necessarily indicate a decline in Ethiopia. There are no nationwide (country-level) studies that show the prevalence and trends of syphilis in Ethiopia. From the global perspective, there is a call to conduct country-level sexually transmitted infection (STI) surveillance and epidemiology estimation [3]. In Ethiopia, there is antenatal care-based sentinel HIV surveillance coordinated and conducted by Ethiopian Public Health Institute (EPHI). The finding of the last four antenatal care-based sentinel HIV surveillance showed inconsistent findings. For instance, the 2007 and 2009 surveillance report showed that syphilis had been declined from 2.7% in 2007 to 2.3% in 2009 [4]. However, the 2012 and 2014 surveillances reports showed a slight increment of syphilis prevalence from 1.0% in 2012 to 1.2 in 2014 [5]. In fact, these antenatal care-based sentinel HIV surveillance estimates included only antenatal care (ANC) clients. There are no pooled data that show the prevalence of syphilis among all and specific subgroups of the population in Ethiopia.

The available individual studies showed a wide variation of prevalence of syphilis among different groups of study population over time and across geographical areas. For instance, syphilis prevalence ranges from 0.1 to 7.5% among blood donors [6–13], 1 to 5.1% among pregnant women [14–21], and 7.3 to 9.8% among HIV patients [22, 23]. Despite the availability of individual studies, a comprehensive synthesis of the available literature that can (i) summarise the national prevalence of syphilis, and (ii) identify and differentiate regional and study population variations is not available.

In order to improve public health responses and allocate resources for syphilis prevention and control, the epidemiology of syphilis in Ethiopia needs to be better characterized. A well organized and synthesized data on the epidemiology of syphilis may help to define target points for syphilis control efforts and thus may help to target interventions to make people towards the elimination of syphilis. It may also help to establish baseline figures against which future trends of syphilis can be monitored in Ethiopia. Thus, this systematic review and meta-analysis will be conducted to synthesize the prevalence of syphilis and summarize geographical and study population differences using the best available evidence.

## Methods

### Settings

This systematic review and meta-analysis will be conducted in Ethiopia. Ethiopia is one of the east African countries located in the horn of Africa and covers a geographical area of 1.1 million km^2^. The country is divided into nine regions and two administrative states, namely, Tigray, Afar, Amhara, Oromia, Somali, Benishangul-Gumuz, Southern Nations Nationalities and People Region, Gambella, Harari, Addis Ababa City administration and Dire Dawa City administration. It is the second-most populous nation on the African continent with over 102 million inhabitants [24].

Syphilis is believed to have been introduced to Ethiopia in the sixteenth or seventeenth century [25]. Gradually, it affects people in all walks of life [25]. It had spread with travelers, migrants, and local population movements, with subsequent local epidemics in native tribes.

Before the establishment of the World Health Organization (WHO), 28,109 syphilitic cases were seen at medical institutions in Ethiopia in 1945/46 [26]. The epidemiology of syphilis had been declining after the introduction of penicillin [27].

Syphilis has got more attention after the emergence of HIV/AIDS as they share the common route of transmission. As a result, syphilis interventions are given along with HIV prevention.

Currently, in addition to behavioral education and communication about sexually transmitted infections, antenatal screening for syphilis to all pregnant women is being implemented in Ethiopia [5].

### Protocol development and registration

We developed this protocol according to the Preferred Reporting Items for Systematic Reviews and Meta-Analysis Protocols (PRISMA-P) 2015 statement [28], and the page numbers where each of the items found in the manuscript are presented in Additional file 1. This protocol has been published in the PROSPERO International Prospective Register of systematic reviews (http://www.crd.york.ac.uk/ PROSPERO), registration number CRD42018116231.

### Criteria for considering studies for review

#### Inclusion criteria

- Studies reporting primary and quantitative data on the prevalence of active syphilis infection will be included. In this review, active syphilis is defined as positive results of at least one of the non-treponemal tests and/or positive results in at least one of the treponemal tests. Furthermore, a positive microscopic observation of *Treponema pallidum* directly from the lesion using microscopic tests (via dark field microscopy) is indicative of active syphilis infection [29, 30]. Non-treponemal tests include rapid plasma reagin (RPR), venereal disease research laboratory test (VDRL), unheated serum test (UST), and toluidine red unheated serum test (TRUST). The treponemal tests include treponema pallidum particle agglutination assay (TPPA), microhemagglutination assay (MHA-TP), treponema pallidum enzyme-linked immunosorbent assay (TP-EIA), and the fluorescent treponemal antibody absorption (FTA-ABS) [29]. The overall interpretation of syphilis is described in Table 1. Lifetime syphilis is defined as negative results in non-treponemal tests and positive results in the treponemal test. Our informal preliminary review of the existing literature showed that further confirmation of negative non-treponemal tests using treponemal tests in Ethiopia is uncommon. As a result, we will not synthesize the prevalence of lifetime syphilis in this systematic review and meta-analysis.
- Articles published only in English. This is because of feasibility issues associated with reading and understanding other languages. Furthermore, reporting articles in other languages is uncommon in Ethiopia. Thus, language bias is minimal in this systematic review.

**Table 1:** Interpretation of serological tests

Non-treponemal test	Treponemal tests	Interpretation
+	+	Active syphilis
+	NC*	
NC*	+	
−	+	Lifetime syphilis/old treated syphilis
+	−	False positive
−	−	No syphilis

*Non-confirmed cases

#### Exclusion criteria

- Studies conducted on patients seeking care for genital symptoms will be excluded to minimize participant selection bias.
- Syphilis diagnosis based on history and physical exam findings will be excluded.
- Studies or reports that include mixed prevalence data on syphilis infection as a composite outcome with another STI and that do not specifically report data on syphilis infection alone.
- For publications that report the same data in multiple sources, the most complete and recent version of the data will be used and others will be excluded.
- Narrative reviews, opinion pieces, letters, case series, and case reports will be also excluded.
- Publications that do not report primary data and/or do not provide a sufficient description of the study methodology.

### Search strategy for identification of relevant studies

We will follow the following two-stage searching strategies:

#### Stage 1: Bibliographic database review

The major electronic databases including MEDLINE/PubMed, EMBASE, Web of Science, CINAHL, The Cochrane Library, Lilacs, and African journal online will be searched for articles published from 1 January 1990 to 1 January 2019. A combination of search terms including “syphilis” or serosyphilis, or “treponema pallidum” or “T. pallidum” or pallidum will be used. In addition, terms that describe the epidemiology of syphilis including prevalence, “seroprevalence,” magnitude and epidemiology will be used. When possible, Medical Subject Headings (MeSH) and/or CINAHL headings will be combined with text words. Otherwise, text words alone will be searched. Reference lists of all included articles will be hand-searched to identify additional studies and reports to include in our analysis. Search strategy for one of the major database is presented in Additional file 2.

#### Stage 2: Gray literature search

Grey literature are documents not published by commercial publishers including agency reports, governmental articles, and academic thesis [31]. To identify grey literature, we use the Google Scholar search engine, official WHO and CDC websites, and the online library of academic and governmental institutions in Ethiopia.

### Selecting studies for inclusion

Articles identified from electronic databases will be exported to Zotero version 5.0.57 for managing references. Using Zotero, duplicated citations will be removed. To select articles based on titles and abstracts, two authors (KM and DD) will apply the inclusion and exclusion criteria to the de-duplicated articles independently. The inter-rater reliability between the two authors (KM and DD) will be calculated using Cohen’s kappa coefficient [32]. Then, articles will be classified into three categories: (1) included, (2) excluded, and (3) pending. Pending articles are articles that initially appear to incompletely fit the exclusion and/or the inclusion criteria.

A decision on the inclusion or exclusion of the pending articles will be made by the fourth authors who had specialization in microbiology (GA). The two authors (KM and DD) will screen the full articles of included articles by applying the inclusion and exclusion criteria listed above. Any disagreement between two authors (KM and DD) during screening and categorization of the full texts will be resolved by the consultation of the third author (AS). All the screening procedures will be presented using the PRISMA flow diagram [33].

### Data collection/extraction

Two authors (KM and DD) will extract data using a pre-designed data extraction format. This data extraction format will be piloted on a randomly selected subsection of studies. Further amendments will be made based on feedback from the pilot testing stage. Two investigators (KM and DD) will extract data independently. Any disagreements in data extraction between KM and DD will be discussed and resolved with the third investigator (AS) acting as arbiter. The investigators will extract data pertaining to the following:

Publication detail—year of publication, first author, and type of publication

Study design**—**study type and sampling method (probability, non-probability)

Study participant—the region where the study took place, study setting (health facility or community), study time frame/study year, type of study participant (pregnant women, general population, HIV, blood donor, etc.), median age, number of the study participant, number/frequency of active syphilis, and diagnostic tools.

Outcome measure**—**a reported estimate of the prevalence of active syphilis in Ethiopia.

### Critical appraisal of included studies

The quality of included studies will be assessed using a 9-item critical appraisal tool adapted from JBI to assess the validity and rate the methodological quality of studies reporting prevalence data [34]. The items used for methodology assessments are presented in Additional file 3.

Two authors (KM and DD) will each independently apply this quality assessment tool to all included studies. Any disagreements between authors will be resolved by consensus discussion and in consultation with the third author (AS).

### Data synthesis including assessment of heterogeneity

The extracted data will be analyzed using Stata software (Stata Corp V.13, TX, USA). Unadjusted prevalence and standard errors of syphilis will be recalculated based on the information of crude numerators and denominators provided by individual studies. As we expect heterogeneity among the included studies, we planned to use a random-effects meta-analysis model. Before applying this model, the variance of the study-specific prevalence will be stabilized with the Freeman-Tukey single arc-sine transformation to keep the effect of studies with extremely small or extremely large prevalence estimates on the overall estimate to a minimum [35].

Heterogeneity among studies will be assessed by the chi-square test on Cochrane’s *Q* statistic [36]. According to Higgins JP and Thompson SG, *I*^2^ values of 25, 50, and 75% represent low, medium, and high heterogeneity, respectively [37].

When there is substantial heterogeneity among studies, we will perform a subgroup analysis to investigate the possible sources of heterogeneity using grouping variables: study region, study population, diagnostic assay/syphilis screening tool, median sample size, year of data collection, study sites, sampling methods, and methodological quality. The presence of publication bias will be assessed using funnel plots and Egger’s test [38]. When there will be publication bias, we will report estimates after adjustment on publication bias using the trim-and-fill method [39].

The inter-rater agreement between investigators for study inclusion, data extraction, and methodological quality will be assessed using Kappa Cohen’s coefficient [32].

### Potential amendments

To avoid the possibility of outcome reporting bias, we will strictly follow the protocol. Any amendments during the review process will be reported transparently.

## Discussion

Despite the availability of individual studies, a synthesized data on the epidemiology of syphilis among all and specific groups of the population is scarce in Ethiopia. The outreaching aim of this review is to synthesize the best available evidence on the epidemiology of syphilis among all and specific groups of the population and to inform policymakers. We will collect both published and unpublished data on the prevalence of syphilis irrespective of the study population. To the best of investigators’ knowledge, this review will be the first systematic review and meta-analysis of the epidemiology of syphilis in Ethiopia.

In Ethiopia, surveillance data on syphilis epidemiology among all and specific groups of the population is lacking except the antenatal care sentinel HIV surveillance [4, 5]. The burden of syphilis needs to be better characterized in terms of different regions and the study population within Ethiopia. The result of this systematic review and meta-analysis may provide robust, valid and reliable data among all and specific groups of the population that supplement the antenatal care sentinel HIV surveillance data.

Understanding the epidemiology of syphilis in all and between specific groups of the population is essential in limed resource countries like Ethiopia to allocate resources and to target specific prevention and control strategies.

In 2019, the adult prevalence of HIV in Ethiopia is estimated to be > 1% (1.13%) [40] and this prevalence is under the category of ‘outbreak of the viruses.’ The prevalence is highest in urban areas (3.0%) [41]. The high level of HIV infection in Ethiopia may also indicate that syphilis infection is still a public health concern in this country. This is because the existence of syphilis worsens the transmission of HIV [1, 42, 43]. Despite this fact, and availability of individual studies, a well synthesized or pooled data on syphilis epidemiology is lacking in Ethiopia.

Based on our informal preliminary review of the existing literature, we expect that there will be a significant difference in the prevalence of syphilis infection among the high-risk population (HIV patients, street dwellers, and factory workers) and low-risk population (pregnant women, elderly and blood donors).

To guide the analysis, we hypothesized that the prevalence of syphilis infection among the high-risk population will be significantly higher than the prevalence of syphilis infection among the low-risk population.

We anticipate that many of study reports are from institutional-based data. As a result, this review would be limited by the predominance of health facility-based studies. This in turn limits the generalizability of findings. In this review, we will include articles published only in the English language. Thus, the introduction of language bias is expected. However, publication in other languages in Ethiopia is uncommon.

We planned to publish the final report of this systematic review in the peer-reviewed journal. Furthermore, findings will be presented at conferences and submitted to relevant health authorities.

## Additional files

Additional file 1: PRISMA-P 2015 Checklist. (DOCX 44 kb)

Additional file 2: Search Strategy for PubMed/Medline. (DOCX 13 kb)

Additional file 3: JBI Critical Appraisal Checklist for Studies Reporting Prevalence Data. (DOCX 12 kb)

## Data Availability

The datasets analyzed during this review will be made available from the corresponding author on reasonable request.

## Abbreviations

AIDS: Acquired immune deficiency syndrome
ANC: Antenatal care
CDC: Center for disease control
HIV: Human immune deficiency virus
MHA-TP: Microhemagglutination assay
PRISMA: Preferred Reporting Items for Systematic Reviews and Meta-Analyses
RPR: Rapid plasma regain
STI: Sexually transmitted infection
TP-EIA: Treponema pallidum enzyme-linked immunosorbent assay
TPPA: Treponema pallidum particle agglutination assay
VDRL: Venereal disease research laboratory test
WHO: World Health Organization
