# Supplementary material for: The epidemiology of syphilis in Ethiopia: a protocol for systematic review and meta-analysis covering the last three decades
Source: Syst Rev. 2019 Aug 22;8:210. doi: 10.1186/s13643-019-1136-z (PMC6706880; doi:10.1186/s13643-019-1136-z)
Supplement: Supplementary file 2 — Search Strategy for PubMed/Medline. (DOCX 13 kb) [file 13643_2019_1136_MOESM2_ESM.docx]

**Additional file 2: Search Strategy for PubMed/Medline**

| Concept | Medical Subject Headings (MeSH) | Text Words | Search strategy |
| --- | --- | --- | --- |
| Syphilis | Syphilis[mesh]  "treponema pallidum"[mesh] | Syphilis[tw]  “Serosyphilis” [tw]  “treponemapallidum”[tw]  [tw]  pallidum [tw] | (((((("Syphilis"[Mesh]) OR "Treponema pallidum"[Mesh]) OR Syphilis[Text Word]) OR “Serosyphilis”[Text Word]) OR “treponemapallidum”[Text Word]) OR “T. pallidum”[Text Word]) OR pallidum |
| Epidemiology |  | “ Sero prevalence” [tw]  Prevalence[tw]  Magnitude[tw]  Epidemiology[tw] | (((“ Sero prevalence”[Text Word]) OR Prevalence[Text Word]) OR Magnitude[Text Word]) OR Epidemiology[Text Word] |
| Ethiopia |  | Ethiopia | Ethiopia[Text Word] |
| Combined search |  |  | ((((((“ Sero prevalence”[Text Word]) OR Prevalence[Text Word]) OR Magnitude[Text Word]) OR Epidemiology[Text Word])) AND ((((((("Syphilis"[Mesh]) OR "Treponema pallidum"[Mesh]) OR Syphilis[Text Word]) OR “Serosyphilis”[Text Word]) OR “treponemapallidum”[Text Word]) OR “T. pallidum”[Text Word]) OR “pallidum”)) AND Ethiopia[Text Word] |
| For CINHAL |  |  |  |
